# Transformer transfer learning emotion detection model: synchronizing socially agreed and self-reported emotions in big data

**DOI:** 10.1007/s00521-023-08276-8

**Published:** 2023-01-26

**Authors:** Sanghyub John Lee, JongYoon Lim, Leo Paas, Ho Seok Ahn

**Affiliations:** 1 Marketing Department, University of Auckland Business School, Auckland, 1142 New Zealand; 2 CARES, Department of Electrical, Computer and Software Engineering, University of Auckland, Auckland, 1142 New Zealand

**Keywords:** Emotion data sets, Emotion detection model, Transformer-based language model, Constructed emotion theory

## Abstract

Tactics to determine the emotions of authors of texts such as Twitter messages often rely on multiple annotators who label relatively small data sets of text passages. An alternative method gathers large text databases that contain the authors’ self-reported emotions, to which artificial intelligence, machine learning, and natural language processing tools can be applied. Both approaches have strength and weaknesses. Emotions evaluated by a few human annotators are susceptible to idiosyncratic biases that reflect the characteristics of the annotators. But models based on large, self-reported emotion data sets may overlook subtle, social emotions that human annotators can recognize. In seeking to establish a means to train emotion detection models so that they can achieve good performance in different contexts, the current study proposes a novel transformer transfer learning approach that parallels human development stages: (1) detect emotions reported by the texts’ authors and (2) synchronize the model with social emotions identified in annotator-rated emotion data sets. The analysis, based on a large, novel, self-reported emotion data set (*n* = 3,654,544) and applied to 10 previously published data sets, shows that the transfer learning emotion model achieves relatively strong performance.

## Introduction

Billions of social networking service users engage in their areas of interest by sharing their opinions and information, mainly as text. Predicting the emotions they express in these texts is critical for both researchers and businesses [31]; consumer emotions expressed in online restaurant reviews [37] or tweets about COVID-19 [18] for example may provide valuable insights for commercial firms and public policy makers. Yet detecting such expressions of human emotions is challenging, particularly when the analyzed data only include text, not facial expressions or other nonverbal information [30].

One option is to task human annotators with generating labels of emotions and categorizing text passages [2, 8, 9, 14, 26, 27, 29, 34]. This stream of research reflects a classical view of emotion theory [35], which postulates that categories such as fear and joy have universal biological fingerprints, inherited by humans through evolution, that get aroused by certain situations. Therefore, annotators should be able to detect emotions expressed in texts authored by others [8, 26, 27, 32]. Yet empirical studies also reveal that the number and categories of emotions vary across individuals, depending their age [11], gender [23], social contexts, and culture [16, 24]. Accordingly, annotators may not be able to judge every emotion expressed by others in all situations, especially those that the annotators have not experienced. In addition, personal experiences are inherently subjective, reflecting the effects of experiential blindness and a person’s current state of mind [5]. For example, one and the same person can be evaluated differently by others, depending on those evaluators’ states of mind. Men who have just crossed a high suspension bridge perceive women as more attractive than men who have not crossed the bridge, because they confuse their fear with attraction [12]. Wang et al. [41] caution that annotators’ assessments of emotions can be subjective and varied too.

Thus an alternative approach analyzes larger data sets, to which emotion labels have been added by the texts’ authors [25, 32, 33], which we refer to as self-rated labels. According to the theory of constructed emotions [6], the human brain uses past experiences, categorized as concepts, to guide people’s actions and give meaning to their observations of their surroundings. Emotions are part of the meaning attributed by and expressed in texts. Data sets with self-reported emotions are relatively easy to collect and can train artificial intelligence (AI), machine learning (ML), and natural language processing (NLP) algorithms [25, 32, 33]. For example, tweets by authors who label them with emotion hashtags, such as #joy, #anger, and #sadness, can be submitted to AI, ML, and NLP models, which produce general emotion detection rules. These rules then can be applied to other tweets that contain emotional content but do not feature labels created by the text’s author or another annotator.

Such ML-based approaches suffer their own limitations; when authors report their own emotions, they might overlook some socially constructed emotions that annotators might be able to identify. Furthermore, models based on self-reported emotion data sets need sufficient amounts of text and labels for training, but what those precise amounts are remains unclear. In particular, ML models do not have previous experience (as annotators might), so they may require more data. Considering these strengths, weaknesses, and gaps, we ask,

- RQ1. Are models based on (1) annotator-labeled or (2) self-reported emotions more accurate in detecting emotions in texts with (a) self-reported or (b) annotated emotion labels?
- RQ2. Does the size of the data set featuring self-reported emotions affect the classification accuracy of ML-based emotion detection models?

Rather than either self-reported emotions or annotated emotion labels, we propose a novel, combined approach, the transformer transfer learning (TTL) model. In this approach, the transformer model gets trained in a series of stages that mimic human development. Over the course of their social-emotional development [22], children first identify their own emotions and then learn to synchronize their emotions with those of others, such as in response to relationship issues [38]. The proposed TTL approach replicates these two stages by first training a transformer model, such as the RoBERTa-large model [21], on a large, self-reported emotion data set, and then on a relatively small, socially agreed emotion data set with annotator-generated labels.

With this novel two-step approach, we aim to improve forecasting accuracy in terms of predicting emotions across different types of data sets, with self-reported or annotator-labeled emotions. To the best of our knowledge, this study is the first to train models sequentially on self-reported emotion data, followed by data gathered from annotator labels. Previous transformer models trained on large emotion data sets achieve higher classification accuracy than those trained on small data sets only [9], though previous research focuses on differences in size of the data sets, not the types of emotion data. We also note a previous study [43] that offers similar findings for chemical reactions, though unrelated to emotion detection.

To confirm whether the newly introduced TTL approach achieves greater emotion detection accuracy than alternative modeling approaches, including non-sequentially trained models that also involve (1) annotator-rated emotion data, (2) self-reported emotion data, and (3) both types, we investigate the following question:

- RQ3. Does the TTL approach achieve higher classification accuracy than models that have been non-sequentially trained on annotator-rated or self-reported emotion data?

In search of answers for these questions, we gather a novel data set with 3,654,544 tweets that include emotion hashtags, inserted by their authors. We analyze this large data set, along with 10 previously published data sets that contain text with emotion labels, whether provided by the texts’ authors or annotators. The new data set can be leveraged for further research; it represents one of the largest data sets of tweets containing self-reported emotion hashtags (*n* = 3,654,544), posted on Twitter between October 2008 to October 2021. It is available as an open data set for academic purposes (https://github.com/EmotionDetection/Self-Reported-SR-emotion-dataset.git).

In turn, the methodological contribution of this paper is threefold. First, we offer the first (to the best of our knowledge) assessment of the generalizability of the forecasting accuracy of emotion detection models developed on the basis of either self-reported or annotator-labeled emotion data sets. To do so, we apply models trained on each self-reported emotion data set to predict the emotions that annotators have used to label the texts and vice versa. Second, regarding the relevance of large databases, we assess whether models developed on the newly collected self-reported data set achieve higher forecasting accuracy than models developed on smaller, previously published data sets that also contain self-reported emotions. Third, we propose and apply the TTL approach, which replicates the social-emotional development stages of children.

## Related work

Growing literature recognizes the importance of specific (fine-grained) emotion detection models and data sets. Existing emotion detection algorithms rely on the concepts of (1) word-level affect lexicon, (2) phrase-level traditional ML, (3) document-level deep learning, and (4) document-level pretrained transformer models.

A word-level affect lexicon entails the identification of a corpus of words related to specific emotions. For example, "stole" relates to the emotion of anger; "amazing" is related to joy. This vocabulary-based approach assigns the primary emotion to a label in the text by searching for the frequency of words associated with various, specific emotions (e.g., [4, 25]).

Phrase-level traditional ML analyzes texts beyond simple counts of words. The algorithms learn the meaning of words or phrases from the training data set, using an *n*-gram function (i.e., sequence of N words; [17]). For example, the 2-g word tokens “fine young” and “young man” can be extracted from the phrase “fine young man” [20, 28, 40].

For document-level deep learning, Goodfellow et al. [15] point out that as the amount of data used for NLP increases, traditional ML algorithms suffer from insufficient lexical feature extraction. Deep learning algorithms instead use many hidden layers to find complex document-level representations of large amounts of text, without lexical feature extraction [1, 26, 32, 41].

In 2018, Google introduced a pretrained transformer model, “Bidirectional Encoder Representations from Transformers” (BERT) [10], that outperformed other models in many NLP tasks, including specific emotion classifications [3, 7, 9]. Previously published studies indicate that transformer models are optimal for emotion detection, but classification accuracy varies depending on the emotion data set being analyzed [3, 7, 9]. As, Table 1 shows, accuracy for classifying human emotions varies from 50 to 80%; it seems difficult to surpass 80%.

**Table 1:** Key reading table for classifying specific emotions

Study	Data	Method	Findings
Balahur et al. [4]	1081 cases	Simple K-means	EmotiNet derived from existing NLP resources, based on the seven most basic emotions (fear, anger, sadness, guilt, shame, joy, and disgust)
Mohammad [25]	21,051 tweets labeled by six emotions	Strength of association (SoA)	Twitter Emotion Corpus (TEC), developed by collecting tweets with emotion hashtags, based on six basic emotions [13]
Mohammad and Kiritchenko [28]	TEC and 1250 newspaper headlines data sets	Support vector machine (SVM)	When using a combination of word-level affect lexicon and (18)-gram function with SVM, the micro F_1_ score was the highest at .499
Liu [20]	TEC data set	Various ML algorithms	The weighted F_1_ score was .579 with SVM, .478 with random forest, and highest at .605 with logistic regression
Volkova and Bachrach [40]	52, 925 tweets	Log-linear models with L2 regularization	Trained the model using lexical features extracted from tweets annotated with six basic emotions, for an overall accuracy score of .78
Abdul-Mageed and Ungar [1]	682,082 tweets	Gated Recurrent Neural Nets (GRNNs) model	The F_1_ score was .8012 on the six basic emotions [13] classification
Wang et al. [41]	Approximately 2.5 million tweets with 131 hashtags	MNB and LIBLINEAR	The F_1_ score was the highest at .6163, with a large-scale linear classification that classifies the seven emotion labels (joy, sadness, anger, love, fear, thankfulness, and surprise)
Saravia et al. [32]	664,462 tweets with 339 hashtags	CNN architecture with a matrix form of the enriched patterns	The average F_1_ score was .79 on eight emotions (sadness, joy, fear, anger, surprise, trust, disgust, and anticipation)
Mohammad et al. [26]	10.983 English tweets for the SemEval-2018 competition	SVM/SVR, LSTMs, and Bi-LSTMs	Deep learning algorithms were applied by the top-performing teams on a classification with four emotions (anger, fear, joy, and sadness)
Chatterjee et al. [7]	41,179 dialogues for the SemEval-2019 competition	Bi-LSTMs with BERT and ELMo	The highest-ranked team achieved a micro F_1_ score of .7959 on four emotions (happy, sad, angry, and others)
Al-Omari et al. [3]	Data set of SemEval-2019	Ensemble model (BERT, GloVe embeddings)	The F_1_ score was .748 on four emotions (happy, sad, angry, and others)
Demszky et al. [9]	58 k Reddit comments	Transformer model, BERT	GoEmotions based on Reddit comments. The macro-average F_1_ score was .64 on the six basic emotions [13] classification

Yet it is not clear whether models based on self-reported emotions generalize to data sets containing annotator labels or vice versa (RQ1). Previous research tends to compare different algorithms used for emotion detection, not the type of data being used to define the emotions expressed in texts. According to this comparison, transformer models outperform alternative algorithms [3, 7, 9], so we integrate them into our proposed TTL approach. Table 1 also shows that emotion data sets exhibit a trend of increasing sizes over time (RQ2) but cannot indicate whether a larger data sets, containing authors’ self-reported emotions, enhance forecasting accuracy.

Finally, recent studies [9, 43] propose that researchers can increase model accuracy by sequentially training a transformer model, first on a large general data set and then on a small, target data set (RQ3). For example, BERT [10] was sequentially trained on a large emotion data set (GoEmotions [9]; *n* = 58 k) and then small data sets (sampled from various emotion data sets, [2] [8] [14] [25] [27] [32] [33]; *n* < 1,000). It achieves greater classification accuracy than models trained only on a small data set [9]. Prior studies include various annotator-rated and self-reported emotion data sets, but the focus is primarily on size differences, rather than types of labels, self-reported or human annotator labels. As noted in the introduction, we aim to extend this approach to replicate the social-emotional development stages of humans and thereby achieve robust performance across emotion data sets.

## Data sets

### Annotator-rated and self-reported emotion data sets

A total of 11 data sets were analyzed, each containing at least four of Ekman's [13] six commonly applied specific emotions (anger, disgust, fear, joy, sadness, and surprise). The data sets consist of emotion labels and accompanying text sentences, such as news headlines, tweets, and Reddit comments. Table 2 summarizes seven previously collected annotator-rated emotion data sets:

- Affective Text (D_A1): Six annotators labeled 1000 news headlines from Google News and CNN (https://web.eecs.umich.edu/~mihalcea/affectivetext/),
- Emotion Cause data set (D_A2): Four annotators labeled 2,000 automatically generated sentences (https://www.site.uottawa.ca/~diana/resources/emotion_stimulus_data/),
- CrowdFlower Sentiment Analysis (D_A3): 40,000 tweets labeled by crowdsourcing (https://data.world/crowdflower/ sentiment-analysis-in-text),
- Emotion Intensities 2017 in Tweets (D_A4): Annotators labeled 7,000 tweets (https://saifmohammad.com/WebPages/EmotionIntensity-SharedTask.html),
- GoEmotions (D_A5): Three to five annotators labeled 58,000 Reddit comments (https://github.com/google-research/google-research/tree/master/goemotions),
- Stance Sentiment Emotion Corpus (SSEC) (D_A6): Three to six annotators labeled 4,800 tweets used in the SemEval 2016 competition (http://www.romanklinger.de/ssec/), and
- SemEval-2018 Affect in Tweets Data (D_A7): Seven annotators labeled 2,500 tweets (http://saifmohammad.com/ WebPages/SentimentEmotionLabeledData.html).

**Table 2:** Annotator-rated emotion data sets

ID	Data set	Ekman emotion	Size	Tot. emotions	Source
D_A1 [2]	Affective text	All 6	1 k	16	News headlines
D_A2 [14]	The emotion cause	All 6	2 k	6	FrameNet
D_A3 [8]	Crowd-flower	Anger surprise joy sadness	40 k	14	Tweets
D_A4 [27]	Emotion intensities 2017	Anger fear joy sadness	7 k	4	Tweets
D_A5 [9]	Go-emotions	All 6	58 k	27	Reddit comments
D_A6 [27, 32]	SSEC	All 6	4.8 k	8	Tweets
D_A7 [26]	SemEval-2018	All 6	2.5 k	11	Tweets

Table 3 lists the three previously published self-reported emotion data sets:

- CARER emotion data set (D_S1): 664,000 tweets with self-reported emotions (https://github.com/dair-ai/emotion_data set),
- International Survey on Emotion Antecedents and Reactions (ISEAR) (D_S2): 7,500 sentences in which participants reported emotions through a survey (https://github.com/sinmaniphel/py_isear_data set),
- Twitter Emotion Corpus (TEC) (D_S3): Collection of 21,000 tweets with emotion hashtags (http://saifmohammad.com/WebPages/SentimentEmotionLabeledData.html).
- Collecting the self-reported emotion data set

**Table 3:** Self-reported emotion data sets

ID	Data set	Ekman emotion	Size	Total emotions	Source
D_S1 [32]	CARER	All 6	664 k	8	Tweets
D_S2 [33]	ISEAR	Joy, fear, anger, sadness, disgust	7.5 k	7	Survey
D_S3 [25]	TEC	All 6	21 k	6	Tweets

We collected the new self-reported emotion data set (D_SR) by using the Twitter Application Programming Interface (API), with approval from Twitter. The collected data set consists of publicly available information and excludes personally identifiable information.

We collected tweets in English (*n* = 5,367,357) posted between March 2008 and October 2021 that feature one of Ekman’s six basic emotions with a hashtag [13]: #anger, #disgust, #fear, #joy, #sadness, and #surprise [25]. For example, the text “Spring is coming!!!!” featured “#joy,” as inserted by the tweet’s author. Emotion hashtags in the tweets (independent variables) affect the dependent variables and were used exclusively as label values. Website addresses and special characters were removed to obtain only English words [25]. For example, exclamation marks were removed from “Spring is coming!!!!,” resulting in “Spring is coming.”

Duplicate tweets also were removed, retaining the first one only (11.32% of tweets in our data set). Tweets including multiple emotion hashtags were removed to capture unique emotion, such as “Everything makes me cry … everything #sadness #angry #joy” [1] (3.95% of tweets). Tweets containing words that may not represent each emotion were also removed, such as #anger together with “management” or “mentalhealth”; #disgust containing “insideout”; #fear in conjunction with “god”; #joy containing “redvelvet”; and #surprise with “birthday” (4.83% of tweets). Also, tweets that had fewer than three English words and re-tweets were excluded [25] (11.81% of tweets). This process resulted in *n* = 3,654,544.

**Fig. 1:**
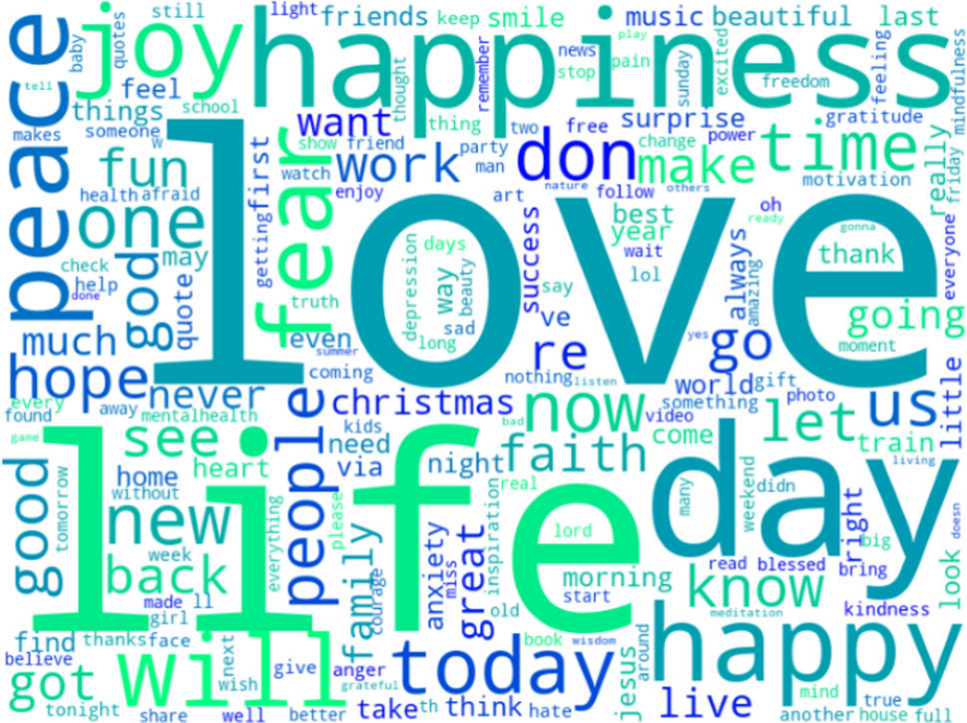
Word cloud of most frequent words

A first descriptive analysis shows that the number of words in a tweet is defined by Mean = 15.59, SD = 8.85, Median = 14, Min = 3, Max = 71, *Q1* = 9, and *Q3* = 20. In Fig. 1, the word cloud of the most frequently occurring words shows that “love” (*n* = 399,484) is the most mentioned emotion word, which reflects general valence. The most mentioned specific emotion is “joy.”

### Integration of eleven emotion data sets

We included only cases reflecting one of Ekman's [13] six emotions (anger, disgust, fear, joy, sadness, and surprise) from the 11 emotion data sets mentioned previously. Table 4 reports the ten previously collected emotion data sets, which contain a total of 108,317 cases that feature four to six Ekman emotions. The new self-reported data set features 3,654,544 cases. The annotator-rated emotion data sets, D_A1 to D_A7, contain a total of 63,516 cases. The previously collected self-reported data sets, D_S1 to D_S3, include 44,801 cases, and D_SR contains 3,654,544 cases. The occurrence of specific emotions across the 11 data sets ranges, defined by proportions from 1.42 to 38.81%.

**Table 4:**
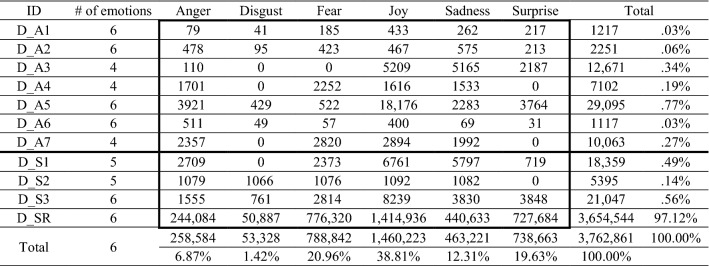
Cases in emotion data sets that contain Ekman emotions

## Methodology

### Transformer models

**Fig. 2:**
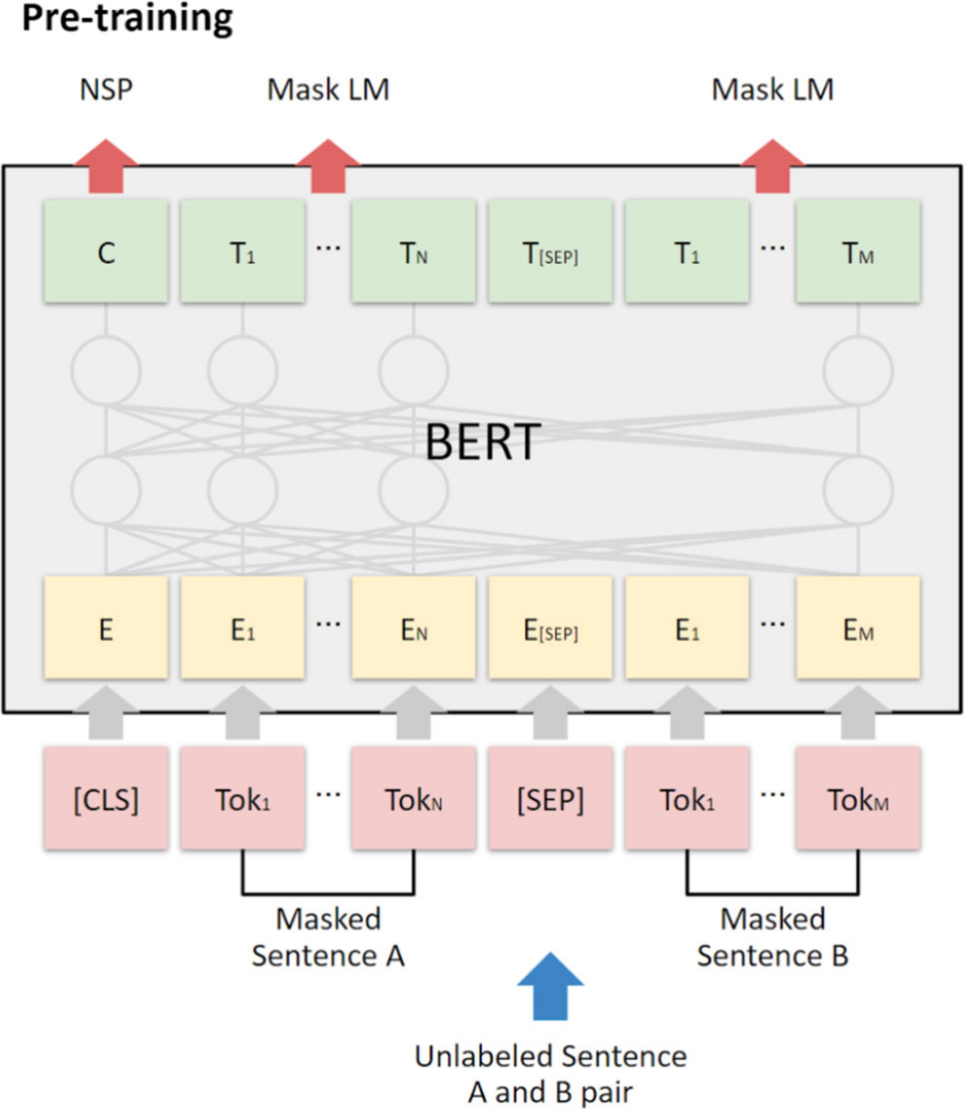
Overview of pretraining BERT [10]

**Fig. 3:**
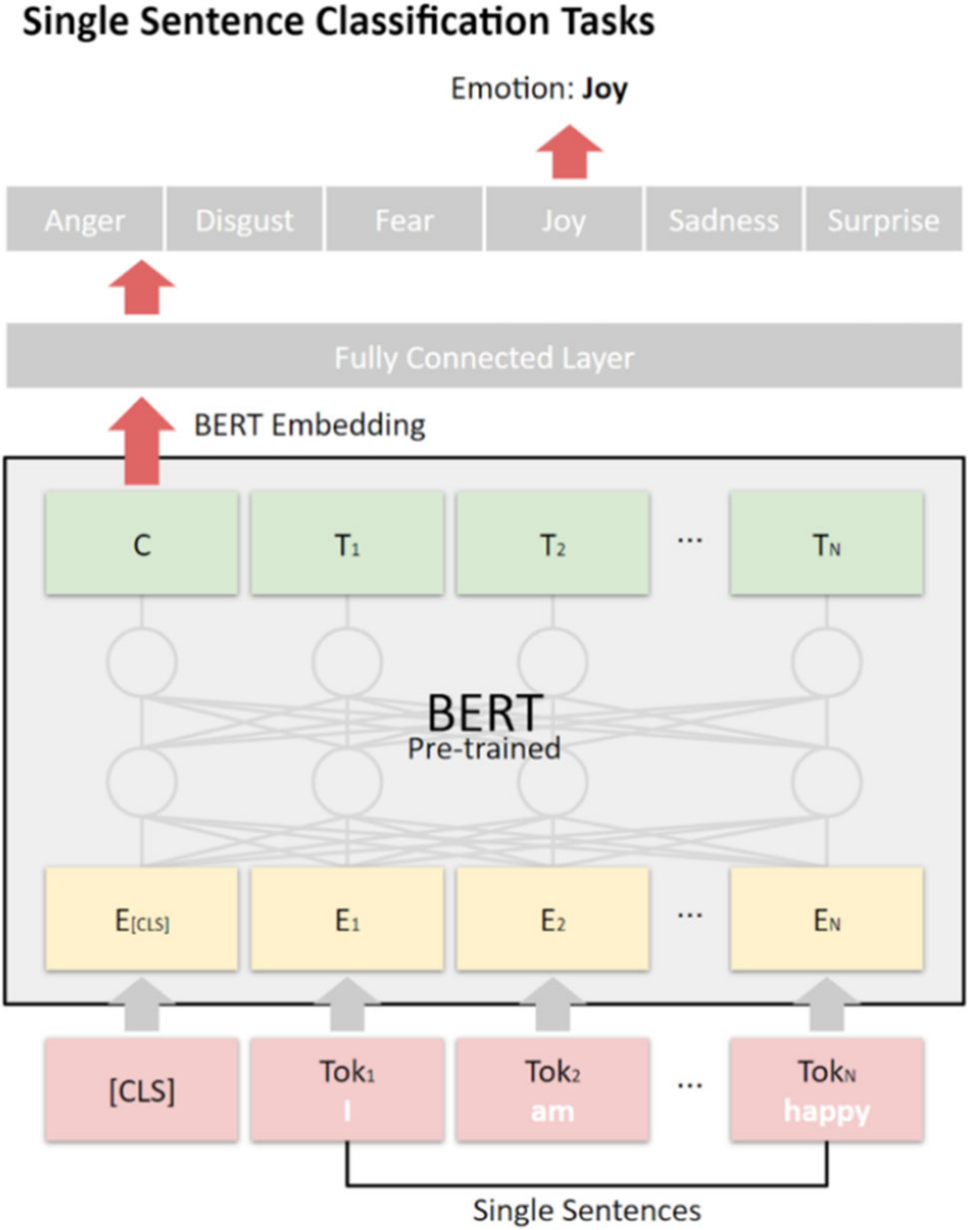
BERT fine-tuning network architecture [10]

As discussed in Sect. 2, transformer models such as BERT [10] outperform alternative ML algorithms [3, 7] for detecting specific emotions in texts. As the first transformer model, BERT relies on text encoders trained on the BooksCorpus (with 800 million words) and the English-language Wikipedia (with 2500 million words) [36]. Its bidirectional training references both left and right sides of sentences simultaneously. Masked language modeling optimizes the weights in BERT model, such that it can train a language model that calculates the probability distribution of words appearing in a sentence on the basis of large, unlabeled texts with unsupervised learning (see Fig. 2). In turn, pretrained BERT models can be fine-tuned with an additional output layer (Fig. 3) to develop high-performing models for a wide range of NLP tasks, such as linguistic acceptability, natural language inferencing, similarity prediction, and sentiment analysis [10]. The fine-tuning process is introduced in more detail in Sect. 4.2.

We also note two versions of BERT: BERT-base (*L* = 12, *H* = 768, *A* = 12, total parameters = 110 M) and BERT-large (*L* = 24, *H* = 1024, *A* = 16, total parameters = 340 M), where L is the number of transformer blocks, H is the hidden size, and A is the number of attention blocks [10]. Because BERT-large contains more training parameters, its training time for a million sentences in our study is prolonged (approximately seven hours per epoch), compared with BERT-base (approximately three hours per epoch), using the latest RTX3090 GPU. Nevertheless, BERT-large achieves better performance than BERT-base.

Liu et al. [21] point out that Robustly optimized BERT approaches (RoBERTa) outperformed BERT in various NLP tasks. This version uses the same architecture as BERT but pretrains ten times more data, including both BERT data and 63 million news articles, a web text corpus, and stories. Because it offers the highest level of forecasting accuracy to date, we apply RoBERTa-large to compare emotion detection models [19].

### Fine-tuning process

**Fig. 4:**
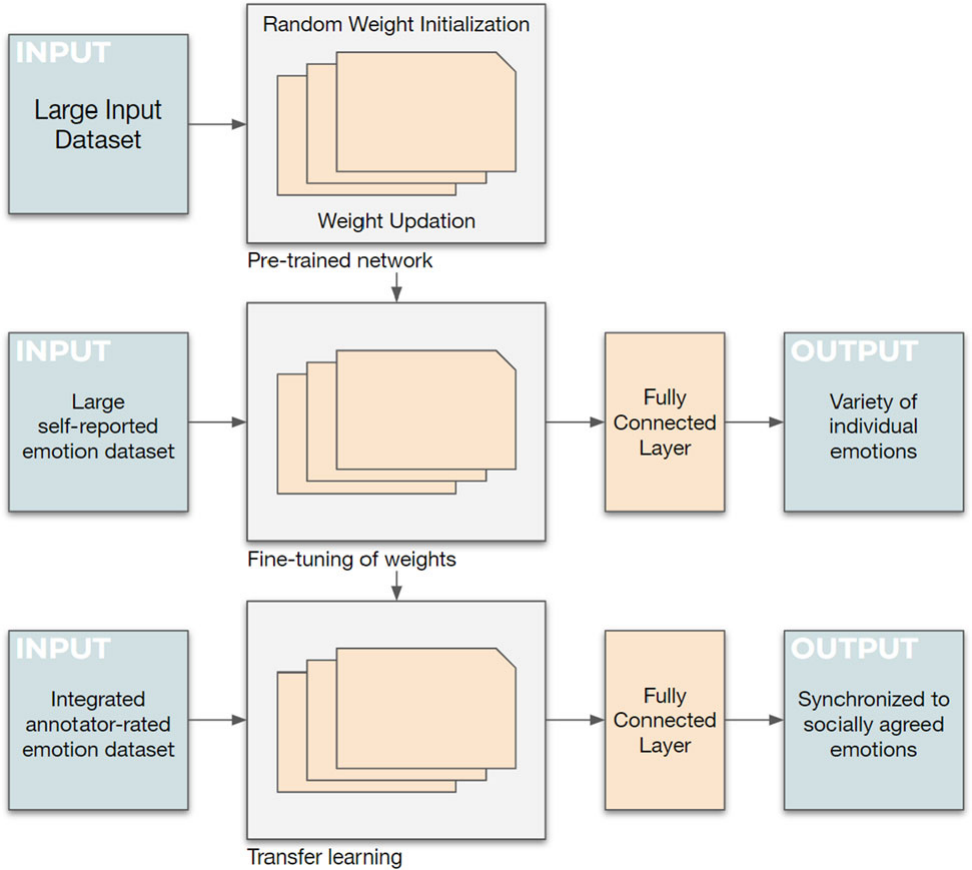
TTL architecture

With RoBERTa, a fine-tuning process takes place for sequence-level classification tasks, as in the BERT architecture. We follow an existing process [19], in which emotion data sets get split into a training (80%) and a testing (20%) data set for the transformer model training. At the beginning of the sequence, a special class token [CLS] gets added for classification tasks. The representation values of dimensions are converted from token values. Because the transformer blocks reflect the relationships of all pairs of words in a sentence, the [CLS] vector indicates the contextual meaning of the entire sentence. In the output layer of the transformer model, we add a fully connected layer to categorize the class labels into specific emotions (Fig. 3). During training, we fine-tuned all parameters of the transformer model together, and the output label probabilities were calculated using the Adam function [19]. This process appears as the middle area in Fig. 4.

Figure 4 summarizes the new two-step TTL architecture that we introduce. First, the transformer model, RoBERTa-large (upper area of Fig. 4), was trained on large self-reported emotion data sets. In our study, the self-reported emotion transformer model (middle area in Fig. 4) was trained on the large, integrated, self-reported emotion data sets, D_S1 to D_S3 and D_SR (*n* = 3,699,345). Second, the emotion model is synchronized to detect socially agreed emotions by retraining on relatively small, annotator-rated emotion data sets. For our study, the model undergoes further training (lower area in Fig. 4) on the combined annotator-rated emotion data sets, D_A1 to D_A7 (*n* = 63,516), through a fine-tuning process, by re-using the self-reported emotion model (middle area in Fig. 4).

The purpose of RoBERTa and other ML models trained with word vectors is to perform a classification in which the output indicates the likelihood of the input sentence being classified as one of the possible emotion labels, such as fear, anger, sadness, joy, surprise, and disgust. We use the weighted F1 score [20] to assess forecasting accuracy; this measure provides a suitable measure of unbalanced data distributions [39], as exist for our analyses. The formula for the F_1_ score of each label (class) can be expressed as follows:

1 $${\mathrm{F}}_{1}\, \mathrm{score}\left(l\right)= \frac{2\times \mathrm{precision}(l)\times \mathrm{recall}(l)}{\mathrm{precision}\left(l\right)+\mathrm{recall}(l)} ,$$

where *l* is the label (anger, disgust, fear, joy, sadness, or surprise), precision(*l*) is $\frac{\mathrm{true positive}(l)}{\mathrm{true positive}(l) +\mathrm{ false positive}(l)}$, and recall(*l*) is $\frac{\mathrm{true positive}(l)}{\mathrm{true positive}(l) +\mathrm{ false negative}(l)}$. To calculate the weighted F_1_ score, we take the mean of all F_1_ scores of each label while weighting the data set size of each label.

## Evaluation

### Evaluation by models trained on separate data sets

To address RQ1 and RQ2, we trained 11 RoBERTa-large models on the 11 separate emotion data sets defined in Table 4. The 11 models take the labels M_A1 to M_A7, M_S1 to M_S3, and M_SR, depending on the specific data set on which they rely. We applied an 80/20 data set splitting strategy to derive a training and a test set from each of the 11 data sets, so for example, the M_A1 model was trained on 80% of the D_A1 data set, and the test set included 20% of D_A1.

Input variables were stored as word vector tokens (pretrained embedding for different words), segment embeddings (sentence number encoded into a vector), and position embeddings (encoded word within the sentence). Output variables consisted of four to six labels, depending on the Ekman emotions available in the data set (Tables 2 and 3).

For the RoBERTa-large models, we used Hugging Face [42], one of the most frequently applied transformer libraries for the Python programming language. The batch size and learning rate are 16 and 0.00001, respectively, in all models. Devlin et al. [10] point out that overfitting may occur after approximately four epochs, due to the transformer model’s numerous parameters, up to 340 million. Thus, training of the RoBERTa-large models was completed in three epochs.

For RQ1, we evaluated the classification accuracy of the seven models based on annotator-labeled data (M_A1 to M_A7) in the seven annotator-rated emotion test sets (D_A1 to D_A7) and in the four self-reported emotion test sets (D_S1 to DS_3 and D_SR). Thus we obtain 77 weighted F1 scores (upper part of Table 5). For example, the first two cells in the top row of Table 5 show that M_A1 produces F_1_ = 0.70 in the 20% test set derived from D_A1, whereas it indicates F_1_ = 0.52 in test set derived from D_A2.

**Table 5:**
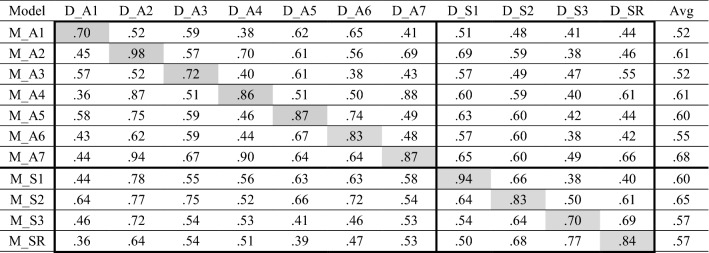
Weighted F_1_ scores of emotion models (M_##) trained on separate data sets (D_##)

The delimiters (D and M) distinguish between data sets (e.g., D_A1) and models (e.g., M_A1). Avg = Average F_1_ score of all test sets; for example, the average value of M_A1 was calculated for D_A1 to D_SR.

The annotator-rated models tend to perform best on test sets derived from the data set on which they were developed, i.e., the diagonal results in Table 5 are relatively high. For example, M_A1 was developed on the training set derived from D_A1, and it achieved an F_1_ score of 0.70 on the test set derived from D_A1—higher than the scores for the ten other test sets (i.e., 0.38 to 0.62). Thus, data sets may be rated by annotators with idiosyncratic rules for labeling emotions.

Table 5 also shows that annotator-rated models (M_A1 to M_A7) resulted in a higher average F_1_ score in test sets of the seven annotator-rated emotion data sets (F_1_ = 0.62; upper left quarter of Table 5) than in the four self-reported emotion test sets (F_1_ = 0.53; upper right quarter of Table 5). For example, in the first row of Table 5, the average F_1_ score of M_A1 is 0.55 for annotator-rated emotion test sets (D_A1 to D_A7) and 0.46 for self-reported emotion test sets (D_S1 to D_S3 and D_SR). The unique rules that annotators apply when specifying emotions in each data set thus appear susceptible to bias in relation to classifying self-reported emotions.

Next, we evaluate the four models trained on the four self-reported emotion data sets, D_S1 to D_S3 and D_SR, in all 11 test sets. The resulting 44 weighted F_1_ scores appear in the lower part of Table 5. Here again, the self-reported models tend to perform best on the test set derived from the data set on which they were developed. We find relatively high diagonal results in Table 5 for the four models developed on the self-reported data sets. Furthermore, the four self-reported models achieve higher average F_1_ scores in self-reported emotion data sets (F_1_ = 0.65; lower right quadrilateral, Table 5) than in annotator-rated emotion data sets (F_1_ = 0.57; lower left quadrilateral, Table 5). Individual authors, across different data sets or in different writing contexts, may exhibit biases similar to those indicated by annotators when expressing their emotions. According to Table 5, this bias has a relatively strong effect when models based on self-reported emotions are applied to data sets with annotator-labeled emotions.

For RQ2, in the self-reported test sets (D_S1 to D_S3 and D_SR), the M_SR model, based on the larger data set, results in the highest average F_1_ score of 0.70, compared with scores from 0.60 to 0.65 for M_S1 to M_S3 (lower right quadrilateral, Table 5). In contrast, the M_SR model achieved the lowest average F_1_ score of 0.49, compared with scores ranging from 0.52 to 0.66 for M_S1 to M_S3, when testing the annotator-rated test sets (lower left quadrilateral, Table 5). Thus, a RoBERTa model trained on large data sets can achieve good performance in similar contexts but not as much in different contexts.

### Evaluation by models trained on multiple data sets

To address RQ3, we used the same input and output variables as in Sect. 5.1. To start, we trained the TTL emotion model on the large, self-reported training set, and then on the smaller, annotator-rated training set. To test the proposed advantages of the TTL approach, we consider three alternative RoBERTa models as benchmarks: (1) the annotator-rated emotion RoBERTa model, trained on the seven annotator-rated training sets (containing 80% of D_A1 to D_A7, *n* = 63,516); (2) the self-reported emotion RoBERTa model, trained on the four self-reported training sets (containing 80% of D_S1 to D_SR, *n* = 3,699,345); and (3) the integration RoBERTa emotion model, trained simultaneously on all 11 data sets, (*n* = 3,762,861), instead of consecutively.

Table 6 contains the 44 weighted F_1_ scores for the TTL emotion model and the three alternatives. The TTL emotion model achieved the highest average F_1_ score of 0.84 across the 11 analyzed data sets. The annotator-rated emotion model achieved the second highest average F_1_ score (0.79).

**Table 6:** Weighted F_1_ scores of emotion models trained on multiple data sets

Model	D_A1	D_A2	D_A3	D_A4	D_A5	D_A6	D_A7	D_S1	D_S2	D_S3	D_SR	Avg
Annotator	.75	.96	.84	.88	.90	.85	.89	.67	.71	.59	.67	.79
Self	.34	.63	.54	.52	.38	.51	.53	.94	.80	.80	.84	.62
Integration	.49	.92	.58	.76	.70	.61	.75	.93	.81	.78	.84	.74
TTL	.77	.97	.83	.88	.90	.83	.90	.89	.79	.68	.79	.84

Annotator = annotator-rated emotion model trained on annotator-rated training sets (D_A1 to D_A7). Self = self-reported emotion model trained on the self-reported training sets (D_S1 to D_SR). Integration = integration emotion model trained on the integrated data sets (annotator-rated and self-reported training sets). TTL = TTL emotion model sequentially trained with self-reported training sets, followed by annotator-rated training sets

**Fig. 5:**
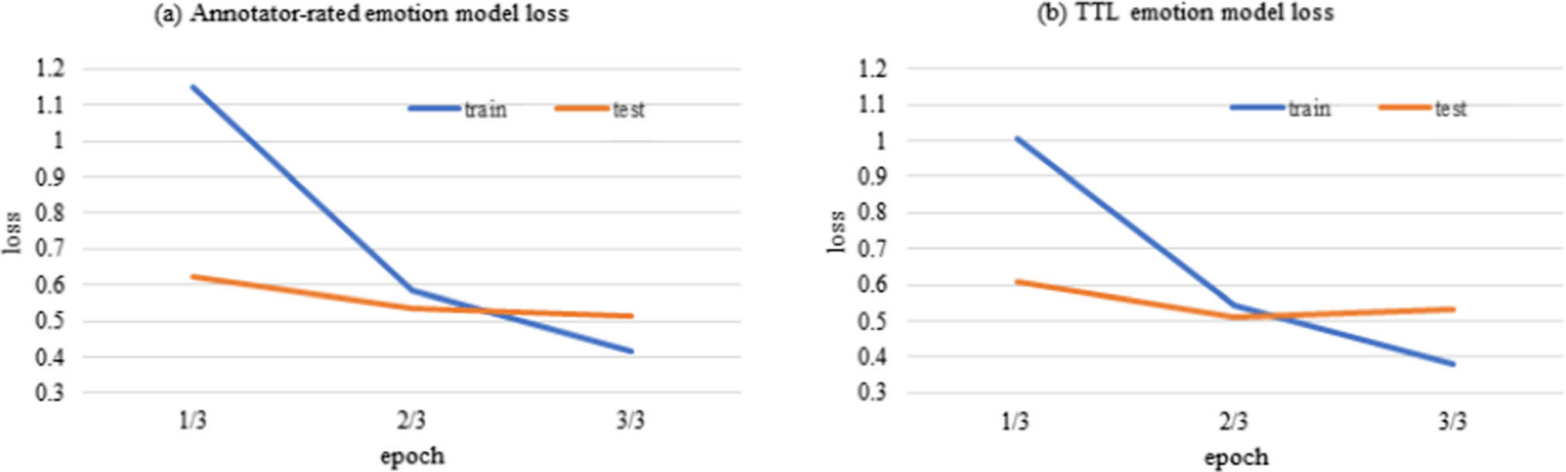
Plots of the losses of **a** annotator-rated emotion model and **b** TTL emotion model during training by annotator-rated training sets

Figure 5 reports the plot of the loss, which reflects the classification error in the training and testing sets that occurs while training annotator-rated training sets. The loss associated with the self-reported emotion model is greater than that linked to the TTL emotion model; that is, the TTL approach can improve the performance of the transformer model during the model training stage. The TTL emotion model achieved the highest (D_A1, D_A5, and D_A7), second highest (D_A2, D_A3, and D_A6), or third highest (D_A4) F_1_ scores in the separate annotator-rated test sets. Furthermore, it achieved above-average F_1_ scores, from fifth (D_S1, D_S2, and D_SR) to sixth (D_S3) highest among of 15 emotion models in the separate self-reported test sets.

**Fig. 6:**
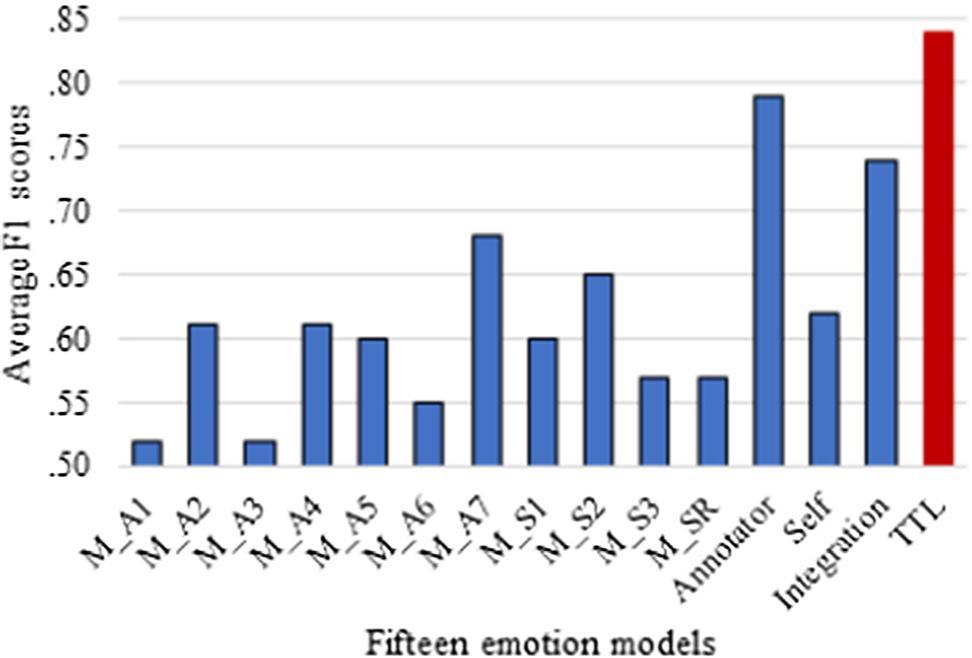
Average F1 scores of fifteen emotion models

Figure 6 plots the average F_1_ scores of the 15 emotion models from Sects. 5.1 and 5.2. The TTL emotion model achieves an average F_1_ of 0.84, which is higher than the values for the 11 models trained on separate data sets (M_A1 to M_SR; F_1_ between 0.52 and 0.68) and three models trained on multiple data sets (annotator-rated, self-reported, and integration emotion models; F_1_ between 0.62 and 0.79).

## Discussion

We examined annotator-rated and self-reported emotion data sets as sources for developing emotion detection models. Each data set has its own rules; any model tends to do best when applied to the test set taken from the data set on which the focal model was trained. This result provides further empirical support for the theory of constructed emotions [6], which argues that the concept of emotion can produce different categories across different people, depending on their personal experiences. This first finding contradicts the classical view of emotion theory [35] that people possess inherent emotions, like universal biological fingerprints.

In relation to RQ1, we find that models developed on annotator-rated emotion data sets perform less well on data sets with self-reported emotions (average F_1_ = 0.53) than on those with annotator-rated emotions (average F_1_ = 0.62). Also relevant for RQ1 is our finding that people are biased in expressing their own emotions, similar to the biases shown by annotators. That is, models developed on self-reported emotion data sets perform less well on data sets with annotator-rated emotions (average F_1_ = 0.57) than on those with self-reported emotions (average F_1_ = 0.65).

For RQ2, the comparison of the findings with models trained on self-reported emotion data sets confirms that the M_SR model trained on a relatively large self-reported data set achieves better performance (average F_1_ = 0.70) in the self-reported emotion test sets than the three models trained on smaller, self-reported emotion data sets (M_S1 = 0.60; M_S2 = 0.65, M_S3 = 0.64). To the best of our knowledge, the D_SR emotion data set is the largest collection of tweets with emotion hashtag labels (*n* = 3,654,544) ever collected, spanning 13 years from October 2008 to October 2021. Nevertheless, M_SR earns a relatively low score on annotator-rated emotion data sets (average F_1_ = 0.49) compared with the other three models that are based on self-reported emotion labels (M_S1 = 0.60, M_S2 = 0.66, M_S3 = 0.64).

To answer RQ3, we offer the TTL emotion model, initially trained on the four combined self-reported emotion data sets (*n* = 3,699,345) and then on the combined annotator-rated emotion data set (*n* = 63,516). The model displays relatively strong performance, with the highest average F_1_ score of 0.84; it achieves the highest average F_1_ score of 0.87 on annotator-rated emotion test sets, but only 0.79 on self-reported emotion test sets. Notably, the TTL emotion model reveals substantial improvements over the annotator-rated emotion models trained on corresponding training sets (D_A1, D_A3, D_A4, D_A5, and D_A7). The average F_1_ score of the TTL emotion model also is higher than those of the integration emotion model that trained all data sets simultaneously, as well as the annotator-rated and self-reported emotion models (Fig. 6).

Further studies might apply the proposed TTL approach to other target domains with small annotator-rated emotion data sets. For example, it might be useful for developing universally applicable emotion detection models that reflect other target domains, such as specific countries (e.g., USA and China), age groups (e.g., children and adults), and genders, based on large, self-reported emotion data sets.

A limitation of this study is that we only collected emotions expressed in tweets, which may not generalize to other text posted on various social media platforms. The TTL emotion model achieved the highest average F_1_ score of 0.84, which is only 0.05 higher than the annotator-rated emotion model value of 0.79. Considering that the classification accuracy of previous emotion detection studies falls between 0.50 and 0.80, it may be difficult to increase the performance of human emotion detection dramatically. Continued research should integrate other types of social media data sets. Also, methods such as ensemble techniques can be used to investigate potential improvements to the accuracy of transformer models.

## Data Availability

The analyzed emotion-labeled data set is shared as an open data set for academic purposes (https://github.com/EmotionDetection/Self-Reported-SR-emotion-dataset.git).
